# Clinical effect of minimally invasive intracranial hematoma in treating hypertensive cerebral hemorrhage

**DOI:** 10.12669/pjms.323.9533

**Published:** 2016

**Authors:** Gang Yang, Gaofeng Shao

**Affiliations:** 1 Gang Yang, Department of Neurosurgery, Zhuji People’s Hospital of Zhejiang Province, Zhejiang, 311800, China; 2 Gaofeng Shao, Department of Neurosurgery, Zhuji People’s Hospital of Zhejiang Province, Zhejiang, 311800, China

**Keywords:** Hypertensive cerebral hemorrhage, Minimally invasive intracranial hematoma, Clinical efficacy, Neurological impairment

## Abstract

**Objective:**

To evaluate the clinical effect of minimally invasive intracranial hematoma in treating hypertensive cerebral hemorrhage.

**Methods:**

One hundred and fifty-six patients with hypertensive cerebral hemorrhage were selected. They were randomly divided into the control group (78 cases) and observation group (78 cases). The control group was treated with conventional craniotomy evacuation of hematoma, while the observation group was treated with minimally invasive intracranial hematoma. Neurological impairment score, treatment efficacy and Barthel index were compared between two groups. Comparison results and clinical data of these patients were retrospectively analyzed.

**Results:**

Neurological impairment score in observation group had a significantly obvious decrease compared to control group (*p* < 0.05). Curative effect of observation group was superior to control group and the difference was significant (*p* < 0.05). Average operation time in observation group (51.20±10.30 minutes) was much shorter than control group (108.60±12.80 minutes). Amount of hematoma cleared for the first time in control group (75.40±10.20 (%)) was more than observation group (45.10±8.70 (%)). Hematoma in observation group (3.90±0.80 days) disappeared faster than control group (5.80±0.90 days). Differences of the above indexes between two groups were all significant (*p* < 0.05). Moreover, Barthel index of observation group was much better than control group (*p* < 0.05).

**Conclusion:**

Treating hypertensive cerebral hemorrhage with minimally invasive intracranial hematoma is remarkably effective. It should be promoted and practiced extensively.

## INTRODUCTION

Hypertensive cerebral hemorrhage is a commonly seen disease in neurosurgery and also one of late complications frequently occurring in hypertension patients.^1^ The disease is induced mostly because of mechanical pressure of hematoma on brain tissue. Hypertensive cerebral hemorrhage with high disability and fatality rate seriusly threatens lives of patients.^2^ A study demonstrates that, annual incidence of hypertensive cerebral hemorrhage was (1 ~ 35)/100 thousand, which accounts for 20% ~ 30% of all stroke patients in Asia.^3^ Mortality and fatality of hypertensive cerebral hemorrhage both rank the first among all stroke types. One-month mortality is between 35% and 52%; most of them die in the early stage; more than 30% survivors still have function impairment; what is worse, its incidence continues to increase with aged tendency of population. Traditional treatment for hypertensive cerebral hemorrhage includes craniotomy evacuation of hematoma and conservative treatment.^4^,^5^ However, conservative treatment and craniotomy evacuation of hematoma cannot achieve ideal curative effect. Thus it is of great importance to treat patient with hypertensive cerebral hemorrhage with effective treatment method.

With the development of imaging technique in recent years, minimally invasive intracranial hematoma has been applied more and more in clinical treatment. Minimally invasive intracranial hematoma as a new technology used for treating cerebral hemorrhage can eliminate hematoma by setting up effective channels for hematoma clearance, without damaging adjacent tissue.^6^,^7^ Compared to traditional craniotomy, minimally invasive intracranial hematoma which can reach hematoma region has little damage to cerebral tissue and can effectively relieve hematoma compression; it is also applicable for patient and even can be used for treating intracranial deep hematoma; moreover, injection of urokinase can accelerate removal of hematoma.^8^ In this study, we selected 156 patients with hypertensive cerebral hemorrhage who received treatment in our hospital between July 2012 and July 2014 as research objects, aiming to discuss our clinical effect of minimally invasive intracranial hematoma in treating hypertensive cerebral hemorrhage.

## METHODS

### General data

Research object of this study was 156 patients with hypertensive cerebral hemorrhage who received treatment from July 2012 to July 2014. They were randomly divided into control group (conventional craniotomy evacuation of hematoma) (78 cases) and observation group (minimally invasive intracranial hematoma) (78 cases). Clinical performance and various examination indexes of patients in two groups all conformed to the clinical diagnostic criteria of hypertensive cerebral hemorrhage formulated in the 4^th^ Cerebrovascular Disease Conference and all patients were confirmed having hypertensive cerebral hemorrhage.^9^ In control group (n = 78), 43 cases were males and 35 cases were females, with age ranging from 30 to 80 years (average 59.77±5.06 years) and bleeding volume ranging from 30 to 180 ml. In observation group (n = 78), 52 cases were males and 26 were females, with age ranging from 32 to 85 years (average 60.18±5.51 years) and bleeding volume ranging from 35 to 180 mL. Gender, age and general condition of patients in two groups had no significant differences (*p* > 0.05); therefore, results were comparable. Patients who suffered from hemorrhage of brain stem, intracranial tumorous hemorrhage or other severe complications or who were in gestation period were not included in this study.

All patients were given conventional treatment such as oxygen supply, correction of water electrolyte balance, intracranial pressure reduction and vital signs monitoring when being admitted into the hospital. Patients in control group underwent conventional craniotomy evacuation of hematoma. Hematoma was removed in direct view after cranium was opened; drainage tube was placed in cavity after surgery was over. Patients in observation group were treated with minimally invasive intracranial hematoma. First, position of hematoma in cranium was confirmed using cranial CT; then position of patient was determined according to haematoma location; site that had the largest area of hematoma and moreover was closest to skull was selected as puncture point; after disinfection, covering with surgical drape and local anaesthesia, skull was punctured; silicone tube was inserted into skull until reaching hematoma region; 10 mL injector was used to slowly absorb hematoma and absorption stopped when there was negative pressure; then drainage tube was inserted; cerebral CT scanning was done in the 1^st^ day after surgery; urokinase (2 ~ 3 thousand units) and 5 mL normal saline were injected, twice each day, and the tube was clamped for once hour after injection; the drainage tube could be removed if patients had no adverse reaction in the 3^rd^ to 7^th^ day after surgery.

### Main observation index and evaluation criteria

Neurological impairment score: neurological impairment was scored before treatment and in the 5^th^, 10^th^ and 15^th^ day after treatment according to clinical neurological impairment scoring criteria of stroke patients formulated by the 4^th^ Cerebrovascular Disease Conference.^10^ If neurological impairment score lowered for 91% ~ 100% and disability was level 0, then patient could be determined as cured. If neurological impairment score reduced to 46% ~ 90% and disability was between level I and level III, then treatment could be considered as significantly effective. If neurological impairment score lowered upto 18% ~ 45%, then treatment was considered as effective; but if neurological impairment score lowered for less than 18%, then treatment was considered as ineffective.

### Barthel index

Barthel index was used to evaluate ten aspects of daily life.^11^ If total score was higher than 80 points, then patient was thought be capable of taking care of himself in most time; if total score was between 35 and 80 points, then patient was considered being able to take care of himself sometimes; if total score was lower than 35 points, then patient was thought incapable of taking care of himself.

### Determination criteria of clinical effect

If hematoma was removed thoroughly and there were no obvious clinical symptoms and vital signs, then treatment was considered to be statistically effective; if hematoma was removed thoroughly and clinical symptoms and vital signs were relieved, then treatment was considered to be effective; if hematoma was not removed thoroughly and clinical symptoms and vital signs had no obvious improvement or even aggravated, then treatment was thought to be ineffective. Overall effective rate = (No. of cases obtaining significant effect + No. of cases obtaining effective treatment) / total number of cases * 100%.

### Statistical analysis

SPSS19.0 was used to process data obtained. Measurement data was expressed as Mean±standard deviation (SD) and processed by *t* test. Enumeration data was processed by chi-square test. Difference was considered to be significant if *p* < 0.05.

## RESULTS

### Comparison of clinical curative effect

In control group, 38 cases (48.72%) were determined having remarkable effect, 25 cases (32.05%)were determined to be effective, and 15 cases (19.23%) were determined to be ineffective; overall effective rate was 80.77%. As to observation group, the corresponding number of cases was 58 (74.36%), 16 (20.51%) and 4 (5.13%); overall efficacy was 94.87%. Clinical effect in observation group was much superior to control group, and the difference was significant (*p* < 0.05). Detailed data are shown in Table-I.

**Table-I:** Comparison of clinical effect [n(%)]

*Group*	*n*	*Remarkable effect*	*Effective*	*Ineffective*	*Overall effective rate*
Control group	78	38(48.72)	25(32.05)	15(19.23)	80.77
Observation group	78	58(74.36)	16(20.51)	4(5.13)	94.87
X^2^					8.895
p					< 0.05

Average operation time, amount of hematoma cleared for the first time and hematoma disappearance time in control group were 108.60±12.80 min, 75.40±10.20 (%) and 3.90±0.80 days; while the corresponding data in observation group was 51.20±10.30 min, 45.10±8.70 (%) and 5.80±0.90 days. The differences were all remarkable. Table-II. Differences of neurological impairment score between two groups before and after treatment were all statistically remarkable (*p* < 0.05). Details are shown in Table-III.

**Table-II:** Comparison of average operation time, amount of hematoma cleared for the first time and hematoma disappearance time between two groups (Mean±SD).

*Group*	*Control group*	*Observation group*	*t value*	*p value*
Average operation time (min)	108.60±12.80	51.20±10.30	12.30	<0.05
Amount of hematoma cleared for the first time (%)	75.40±10.20	45.10±8.70	15.12	<0.05
Hematoma disappearance time (days)	5.80±0.90	3.90±0.80	10.07	<0.05

**Table-III:** Comparison of neurological impairment score between two groups (Mean±SD).

*Group*	*n*	*Before*	*After treatment*		

			*5d*	*10d*	*15d*
Control group	78	13.40±5.12	13.60±6.05	12.30±5.34	9.89±3.31
Observation group	78	14.60±9.72	10.90±4.30	8.85±4.52	3.20±2.95
t		0.9676	3.0544	6.5921	10.5781
p		>0.05	<0.05	<0.05	<0.05

Barthel index of patients in control group and observation group before treatment was lower than 36 points, and the difference was insignificant. But in the 4^th^ and 12^th^ week after treatment, patients with Barthel index higher than 35 points accounted for a large proportion in observation group, and the difference was remarkable. Table-IV.

**Table-IV:** Comparison of Barthel index between two groups before and after treatment.

*Group*		*<35 points*	*35~80 points*	*>80 points*
Control group (n=78)	Before	47(60.26)	31(39.74)	0(0.00)
	4th week after treatment	31(39.74)	41(52.57)	6(7.69)
	12th week after treatment	19(24.36)	44(56.41)	15(19.23)
Observation group (n=78)	Before	47(60.26)	31(39.74)	0(0.00)
	4th week after treatment	15(19.23)*	44(56.41)	19(24.36)
	12th week after treatment	3(3.85)*	44(56.41)	31(39.74)

* means p < 0.05 compared to control group.

## DISCUSSION

Incidence of high blood pressure associated complications increases with increase in high blood pressure. Hypertensive cerebral hemorrhage as one of the severe complications of patients with high blood pressure is more likely to induce disability and death; about 35% ~ 52% patients with hypertensive cerebral hemorrhage die within 30 days after onset and only 20% patients can take care of themselves in 6 months after onset.^12^ Previous studies demonstrate that, main causes for hypertensive cerebral hemorrhage include micro-aneurysm formed in cerebral arteriole at basal ganglion, weakened structure of external membrane and middle-layer membrane of cerebral arterial wall, spasm of cerebral arteriole induced by high blood pressure and fibrinoid necrosis of cerebral arteriole induced by high blood pressure.^13^,^14^ Therefore treatment timeliness of hypertensive cerebral hemorrhage is required high. In previous studies, drug treatment is used for treating hypertensive cerebral hemorrhage, but with poor curative effect. Before application of CT, mortality of medicinal treatment was between 70% and 80%.^15^,^16^ Traditional craniotomy which can remove hematoma relatively thoroughly is applied afterwards.^17^ But it has several disadvantages including big trauma, obvious impairment on cerebral tissue, long operation time, multiple complications and poor prognosis and its curative effect is difficult to be improved even when indications are strictly limited. It has been reported that, mortality of patients with hypertensive cerebral hemorrhage who undergo traditional craniotomy is about 50%, which is close to drug treatment.^18^ It is seldom used now except for some special situations.

Now small bone window hematoma evacuation is frequently used. Small bone window hematoma evacuation, an improvement of conventional craniotomy evacuation of hematoma, removes hematoma by cutting incision in site with hematoma in the shallowest site and smallest damage and removing bone window. It is suitable for cerebral hemorrhage in cortex or closing to cortex and is a good operation method currently. But it is not applicable for removing hemorrhage in deep site. Thus it is necessary to find out a new method which is effective and safe for hypertensive cerebral hemorrhage.

In recent years, studies on application of minimally invasive intracranial hematoma in hypertensive cerebral hemorrhage has increased.^19^,^20^ Most are positive, but some show low level of recognition. Hence it is great value to research application of minimally invasive intracranial hematoma in hypertensive cerebral hemorrhage. Minimally invasive intracranial hematoma with advantages of simple operation, small trauma and short operation time is an effective method for treating hypertensive cerebral hemorrhage.^21^,^22^ In this study, we observed clinical effect of minimally invasive intracranial hematoma in treating hypertensive cerebral hemorrhage and compared it with conventional craniotomy evacuation of hematoma. Results suggested that, overall efficacy in observation group (94.87%) had a significant difference with control group (80.77%), and moreover average operation time, hematoma disappearance time and amount of hematoma clearance for the first time in observation group were all superior to control group, indicating minimally invasive intracranial hematoma is effective in treating hypertensive cerebral hemorrhage.

In addition, we also found that, improvement of neurological function of patients receiving minimally invasive intracranial hematoma was much obvious than those who received conventional craniotomy evacuation of hematoma, which confirms the short-term effect of minimally invasive intracranial hematoma; Barthel index of patients in observation group showed continuous improvement at different time points, which also confirms continuous improvement effect of minimally invasive intracranial hematoma. These are all associated with rapid and effective hematoma clearance of minimally invasive intracranial hematoma. As removing hematoma with puncture suction is rapid, minimally invasive and safe, neurological impairment induced by hematoma can be relieved rapidly and effectively, which is an important basis and improves prognosis of patients. What is more, improvement of neurological function can lay a basis for improvement of living capability of patients in the future. Therefore, its comprehensive advantage is outstanding.

In the perspective of neurological impairment score, observation group had a rapid decline compared to control group, suggesting patients’ benefits from minimally invasive treatment. That is because timely removal of hematoma eliminates space occupying effect of hematoma, blocks initial factors that can induce edema and prevents secondary injury, thereby protecting survived cerebral tissue to the largest extent.

## CONCLUSION

To sum up, minimally invasive intracranial hematoma with advantages of simple operation, small trauma and high efficiency can effectively improve neurological function of patients with hypertensive cerebral hemorrhage. It is safe and reliable, thus can be promoted and practiced clinically.
